# Changes in transcriptome of native nasal epithelium expressing F508del-CFTR and intersecting data from comparable studies

**DOI:** 10.1186/1465-9921-14-38

**Published:** 2013-03-28

**Authors:** Luka A Clarke, Lisete Sousa, Celeste Barreto, Margarida D Amaral

**Affiliations:** 1 BioFIG - Centre for Biodiversity, Functional and Integrative Genomics; FCUL -Faculty of Sciences, University of Lisboa, Lisboa, 1749-016, Portugal; 2 CEAUL - Centre of Statistics and Applications; FCUL - Faculty of Sciences, University of Lisboa, Lisboa, 1749-016, Portugal; 3 Department of Pediatrics, Hospital de Santa Maria, Avenida Professor Egas Moniz, Lisboa, 1649-035, Portugal

## Abstract

**Background:**

Microarray studies related to cystic fibrosis (CF) airway gene expression have gone some way in clarifying the complex molecular background of CF lung diseases, but have made little progress in defining a robust “molecular signature” associated with mutant CFTR expression. Disparate methodological and statistical analyses complicate comparisons between independent studies of the CF transcriptome, and although each study may be valid in isolation, the conclusions reached differ widely.

**Methods:**

We carried out a small-scale whole genome microarray study of gene expression in human native nasal epithelial cells from F508del-CFTR homozygotes in comparison to non-CF controls. We performed superficial comparisons with other microarray datasets in an attempt to identify a subset of regulated genes that could act as a signature of F508del-CFTR expression in native airway tissue samples.

**Results:**

Among the alterations detected in CF, up-regulation of genes involved in cell proliferation, and down-regulation of cilia genes were the most notable. Other changes involved gene expression changes in calcium and membrane pathways, inflammation, defence response, wound healing and the involvement of estrogen signalling. Comparison of our data set with previously published studies allowed us to assess the consistency of independent microarray data sets, and shed light on the limitations of such snapshot studies in measuring a system as subtle and dynamic as the transcriptome. Comparison of *in-vivo* studies nevertheless yielded a small molecular CF signature worthy of future investigation.

**Conclusions:**

Despite the variability among the independent studies, the current CF transcriptome meta-analysis identified subsets of differentially expressed genes in native airway tissues which provide both interesting clues to CF pathogenesis and a possible CF biomarker.

## Introduction

Cystic Fibrosis (CF) is a clinically complex disease [1] caused primarily by mutations in the cystic fibrosis transmembrane conductance regulator (CFTR) gene [2], which encodes a chloride (Cl^-^) channel that plays a fundamental role in ion and fluid transport across epithelial surfaces [3]. The CF phenotype depends greatly on what combination of mutant CFTR alleles is present out of the more than 1,900 currently listed (http://www.genet.sickkids.on.ca/StatisticsPage.html) [4]. F508del-CFTR [5], which accounts for up to 90% of CF alleles [6], is associated with a severe clinical phenotype, but even F508del-homozygous CF patients display much phenotypic heterogeneity [7]. Although such heterogeneity can partly be explained by genetic modifiers [8-12] or environmental factors [13,14], it is desirable to determine how F508del-CFTR specifically affects global gene expression, in order to clarify how a dynamic network of interactions surrounding CFTR at the cellular level [15] is perturbed in the most widespread form of CF.

Several CF transcriptomics studies have employed microarrays to measure differences in global gene expression caused by the F508del mutation in isogenic bronchial cells [16] (in this case the CFTR genotype was F508del/W1282X), primary cultures of tracheal and bronchial cells [17], native nasal epithelial and bronchial cells [18,19] and immortalized foetal tracheal cell lines [20]. Two of these studies [16,20] used technical replicates of the same source material, thus avoiding the problem of individual variation present in studies using biological replicates, but also reducing their interest as general models of F508del-CFTR related gene expression. Studies on native tissues have reported differential expression of genes involved in a variety of cellular processes relevant to CF, such as airway defence and mitochondrial function [18], or inflammation and cellular movement [19]. In contrast, similar work in primary cultures of epithelial cells from CF patients, led to the conclusion that F508del-CFTR had a minimal effect on global gene expression [17], suggesting that the differences found in native cells were secondary. Studies focusing on expression differences associated with severity of CF phenotype [18] and others on expression patterns of nasal vs. bronchial epithelium [19] also produced widely differing patterns of global gene expression. A recent meta-analysis of four independent microarray studies [21] concluded that very few individual genes were among the highest regulated in more than two of the four studies, and that there was little evidence associating induction of pro-inflammatory pathways with the presence of F508del-CFTR.

Herein, we present the results of a small-scale microarray study of differential gene expression in human native nasal epithelial cells from five F508del-homozygous CF patients *vs*. five control individuals. Data analysis using the Rank Products (RP) method resulted in a list of differentially expressed genes, many of which are functionally relevant, given our knowledge of CF. Some others, although not previously connected to CF, fall into enriched gene ontology (GO) groups relevant to CF, including cell proliferation, calcium binding, plasma membrane and cilium. A comparison of our data with gene lists obtained in five similar studies led us to conclude that the genes shared between independent gene lists did not constitute a robust molecular signature, although many of the genes shared were highly relevant to CF. However, reanalysis of the results of a recent study of CF related gene expression in bronchial and nasal epithelium [19] followed by direct comparison with our own dataset led to the identification of a small subset of regulated genes as a putative gene signature characteristic of the CF airway.

## Materials and methods

### Participant selection and nasal respiratory epithelial cell collection

The study was conducted at the Faculty of Sciences of the University of Lisboa with samples collected at the CF Clinic of the Department of Pediatrics of the Hospital de Santa Maria in Lisboa, and was approved by the Santa Maria Hospital Ethical Review Board. Informed consent was obtained from each participant, or parent/tutor where the participant was a minor. To be eligible for the study, individuals with CF had been previously confirmed to be homozygous for the F508del mutation. Individuals with recent viral infection, or active CF exacerbation were excluded. Non-CF control subjects were recruited from overweight but healthy teen-aged volunteers attending the paediatric clinic for check-ups related to weight loss. If participants demonstrated obvious turbinate inflammation or haemorrhage on initial brushing, the sample was excluded. Participants’ data including parameters of lung function can be found in Table 1. Bilateral nasal mucosal brushing to collect respiratory epithelium was performed on each subject as described before [22,23]. An aliquot was set aside for cytological evaluation, and the remainder frozen at −80°C prior to RNA extraction.

**Table 1:** Identity of nasal epithelial cell samples used in microarray analysis

**Individual**	**Gender**	**Age (yr)**	**% inflamm. cells**	**FVC (% of expected)**	**FEV1 (% of expected)**
**CF** (*F508del homozygous*)					
**BC**	F	9	5.1	1.70 L (88.7%)	1.39 L (84.6%)
**FC**	M	17	9.8	4.16 L (90.6%)	3.36 L (88.5%)
**JF**	M	16	6.5	2.65 L (60.7%)	1.43 L (39.7%)
**PM**	F	10	4.2	0.60 L (38.3%)	0.60 L (43.8%)
**SS**	F	18	4.3	3.00 L (78.5%)	2.28 L (70.7%)
***Mean***	-	*14*	*5.98*		
**non CF**					
**AC**	F	16	1.6	n/a	n/a
**BR**	M	16	3.7	n/a	n/a
**FR**	M	14	4.4	n/a	n/a
**JR**	M	14	8.1	n/a	n/a
**SP**	F	14	5.7	n/a	n/a
***Mean***	-	*14.8*	*4.7*		

Neither age (p = 0.69, student’s t test) nor percentage of inflammatory cells per sample (p = 0.42, student’s t test) showed any significant difference between groups. Data on lung function were obtained for CF patients only: percentage of expected function for relevant age group shown in brackets.

### Cytological evaluation

A cytospin centrifuge (Shandon, Thermo Scientific, USA) was used to prepare formaldehyde-fixed respiratory epithelial cell samples for standard May-Grünwald-Giemsa (MGG) staining, as previously described [24]. Identity of samples was obscured with randomly numbered labels for blind counting. Slides were then evaluated twice using light microscopy and digital photography of 5 high power fields of view per sample, and cells were categorized as epithelial or inflammatory. Results were expressed as the percentage of total cells (Table 1). Samples with more than 10% inflammatory cells were excluded from the study. The data obtained agreed with results from previous studies, in which approximately 5-10% of cells from nasal brushing have been shown to be of inflammatory origin regardless of CF status [18,24].

### RNA isolation, target synthesis and hybridization to AffymetrixGeneChips

Total RNA was extracted using the RNeasy Mini Kit (Qiagen, Hilden, Germany). Concentration and purity was determined by spectrophotometry (Nanodrop) and integrity (RIN > 7.0: mean RIN, CF - 8.2; Control - 8.4) was confirmed using an Agilent 2100 Bioanalyzer with a RNA 6000 Nano Assay (Agilent Technologies, Palo Alto, CA). Five CF and five control RNA samples were chosen for the final microarray hybridization. RNA was processed for use on Affymetrix (Santa Clara, CA, USA) GeneChip HsAirwaya520108F Arrays, which were custom-designed to determine gene expression in the human airway epithelium [25], according to the manufacturer’s Two-Cycle Target Labelling Assay. Briefly, 90 ng of total RNA containing spiked in Poly-A RNA controls (GeneChip Expression GeneChip Eukaryotic Poly-A RNA Control Kit; Affymetrix) were used in a reverse transcription reaction (Two-Cycle DNA synthesis kit; Affymetrix) to generate first-strand cDNA. After second-strand synthesis, double-stranded cDNA was used in an *in vitro* transcription (IVT) reaction to generate cRNA (MEGAscript T7 kit; Ambion, Austin, TX). 600 ng of the cRNA obtained was used for a second round of cDNA and cRNA synthesis, resulting in biotinylated cRNA (GeneChip Expression 3^′^-Amplification Reagents for IVT-Labeling; Affymetrix). Size distribution of the cRNA and fragmented cRNA, respectively, were assessed using an Agilent 2100 Bioanalyzer with a RNA 6000 Nano Assay. 15 μg of fragmented cRNA was used in a 300-μl hybridization containing added hybridization controls. A final volume of 200 μl was hybridized on arrays for 16 h at 45°C. Standard post hybridization wash and double-stain protocols (EukGE-WS2v5) were used on an Affymetrix GeneChip Fluidics Station 400. Arrays were scanned on an Affymetrix GeneChip scanner 3000.

### Microarray data analysis

Genechip expression data were quantile normalized in RMA Express [26], following examination of QC parameters (GAPDH ratios, log_2_PM distributions and RLE/NUSE plots). Normalized values were then analysed using the Rank Products method (Bioconductor Package RankProd). Rank Products (RP) is a non-parametric method used to detect genes that are consistently highly ranked (strongly up-regulated/down-regulated between two conditions), particularly in experiments with a small number of replicates where it has been shown to generate accurate results [27,28]. The null hypothesis assumes that the order of all genes is random, thus the RPs are compared with the RPs for 1000 random permutations, with the same number of replicates and genes as the real experiment in order to correct for the multiple testing problem inherent in microarray experiments. To assign a significance level, the associated p-value and the false discovery rate (FDR) are included in the output alongside the genes that are detected by using certain criteria. This method has been used in various application domains, including proteomics, metabolomics, statistical meta-analysis, and general feature selection [29-31]. The gene list thus ranked according to the RP statistic can be further organised according to p value and FDR. A dendrogram showing how the individual array experiments clustered was also generated in BRB *Arraytools*[32], using centred correlation and average linkage. A MIAME-compliant microarray data submission [33] was made to the Gene Expression Omnibus (https://www.ncbi.nlm.nih.gov/geo/, accession number GSE40445).

### Pathway and GO analysis

A reduced RP-ranked gene list was produced from the analysed microarray data by using a p-value cut-off of 0.0001 followed by removal of any gene with FDR > 0.05 and addition of a detection call filter (>20% present in all samples). This resulted in a list comprising 133 up-regulated and 255 down-regulated probesets (CF/Control: Additional file 1). This list was then submitted to the DAVID functional annotation tool [34,35] (http://david.abcc.ncifcrf.gov/). This software generates a list of GO terms (classified as Biological Process, Cellular Compartment or Molecular Function) found to be enriched (ie, non-randomly distributed) in the submitted gene list. The same list was also used in pathway discovery using the GeneGo Metacore® platform (http://thomsonreuters.com/products_services/science/systems-biology/). Gene Set Enrichment Analysis [36] was used to assess the distributions in our data set of regulated genes (used as gene sets) from five comparable studies [16-20], and gene sets relevant to the previous analysis from the molecular signature database of the GSEA website (http://www.broadinstitute.org/gsea/msigdb/index.jsp). GSEA software provides an enrichment score (ES) and a p value to assess whether a given gene set is preferentially associated with one end of a data set, meaning that expression of the genes in a given gene set is associated with one of the phenotype groups under study.

### Quantitative real time PCR

Sequences for quantitative real time (qRT-) PCR primers were found at Harvard Primerbank [37] (HP: http://pga.mgh.harvard.edu/primerbank/) (Table 2) or, if HP primers proved unsuitable, additional pairs were designed using Primer3 software (http://frodo.wi.mit.edu/). All primer pairs were tested to ensure product specificity, and amplification efficiencies were determined for each primer pair using standard curves by amplification of a series of 1:5 dilutions of pooled control nasal cell cDNAs. Primer pairs with efficiencies less than 90% or more than 110% were not used. Estimates of differential gene expression relative to expression of *ACTB* and *GAPDH*, two housekeeping genes, were performed for 10 genes chosen from the microarray gene lists in PCR reactions containing forward and reverse primers (0.25 μM each), cDNA (approximately 5 ng) and 1 x EVAGreen PCR Master Mix (BioRad) in a 20 μl reaction volume, using a Cx96 PCR machine (BioRad). The Pfaffl method of relative quantification [38] was used to compare expression of test genes in CF and non CF samples, normalized against expression of the control genes (*ACTB* and *GAPDH*). A standard cycle protocol was used for PCR amplification (1 min at 95°C followed by 40 cycles of 10 sec at 95°C and 30 sec at 60°C, followed by production of a 60°C to 95°C melt curve in increments of 0.5°C), and fluorescence data was collected using the CFX software suite (BioRad). Means of *C*_T_ values from 3 technical replicates of each gene were used in calculating the normalized fold change (FC) between CF and normal tissues, using the Pfaffl formula (FC = (E*target*^)ΔCT*target*(control-CF)^/(E*ref*)^ΔCT*ref*(control-CF)^) where *FC* is the fold change, *E* is primer efficiency, and *target* and *ref* are the test and reference genes, respectively. Real Time PCR was used for measurement of relative gene expression in 6 x CF (average age 12.0 years; 1 M, 5 F; average RIN 9.3) and 5 x Control (average age 13.4 years; 1 M, 4 F; average RIN 9.0) nasal cell samples collected independently and treated according to the methods described above.

**Table 2:** Primers used in qRT-PCR amplification

**Official gene symbol**	**Primer sequences (5**^**′ **^**to 3**^**′**^**)**	**Primerbank ID**	**Product size**	**Efficiency**
ACTB (Ref. Gene)	Fwd: CTCTTCCAGCCTTCCTTCCT	N/A	116 bp	100%
	Rev: AGCACTGTGTTGGCGTACAG			
ADM	Fwd: ATGAAGCTGGTTTCCGTCG	4501945a1	112 bp	100%
	Rev: GCCCACTTATTCCACTTCTTTCG			
AQP9	Fwd: CTGCAACCGTCTTTGGCATTT	10280624a3	118 bp	106%
	Rev: AGATACGGAGCTGGGTATGTT			
AREG	Fwd: GTGGTGCTGTCGCTCTTGATA	4502199a1	171 bp	97%
	Rev: ACTCACAGGGGAAATCTCACT			
GAPDH (Ref. Gene)	Fwd: CATGAGAAGTATGACAACAGCCT	7669492a3	113 bp	97%
	Rev: AGTCCTTCCACGATACCAAAGT			
GJA1 (Connexin 43)	Fwd: GTGCCTGAACTTGCCTTTTC	N/A	165 bp	98%
	Rev: CCCTCCAGCAGTTGAGTAGG			
IGFBP3	Fwd: AGAGCACAGATACCCAGAACT	4504617a2	105 bp	100%
	Rev: TGAGGAACTTCAGGTGATTCAGT			
NDRG1	Fwd: TCGAGACTTTACATGGCTCTGT	207028746b2	93 bp	106%
	Rev: TCATGCCGATGTCATGGTAGG			
SCGB1A1 (Uteroglobin)	Fwd: TTCAGCGTGTCATCGAAACCC	4507809a1	189 bp	100%
	Rev: ACAGTGAGCTTTGGGCTATTTTT			
SPAG6	Fwd: GTAAGGTGCTGCCGCATGATA	6912678a1	152 bp	100%
	Rev: CCTCACTATTTCCTCGGGGTA			
TEKT1	Fwd: CAGATTCGGATGAACCGCTCT	16753231a3	140 bp	103%
	Rev: CTCACGGCGTTCTCAGAATATC			
TMEM45a	Fwd: GTTCACTTCCTGTGTCCTTAACC	8922242b2	95 bp	97%
	Rev: CATTTCCCGGCCATGAGTGT			

Forward and reverse primers for amplification of the indicated genes were found at Harvard Primerbank (primer IDs shown; N/A indicates in-house primer design). Product sizes and primer efficiencies for Pfaffl calculations are also shown.

### Comparison with published gene lists

In order to find regulated genes in common between independent studies, we compared our lists of up- and down-regulated genes with gene lists from the four studies [16-18,20] previously reanalysed [21] and which were kindly provided by the authors (T.H. Hampton & B.A. Stanton, *pers. comm*.), and from a more recent paper [19]. The 4 reanalysed lists [21] were generated using an exploratory gene array analysis method to find the optimal number of genes for pathway analysis (set at 300 genes) by using different p values and differential expression cut-offs for each data set. The 4 data sets in that meta-analysis have also been reduced to a set of 8,858 genes common to the 4 array platforms used. Although 426 genes were found to be up-regulated in CF-vs.-non CF bronchial epithelial cell samples by Ogilvie et al. [19], we used the most stringently defined list provided in the supplementary data section of that paper (cut-off set at fold change of +/− 2, and p < 0.05) resulting in lists of 115 up- and 110 down-regulated genes for this preliminary comparison. For direct comparison of our gene lists with the 5 other studies [16-20], all gene lists were converted into official gene symbols (http://www.ncbi.nlm.nih.gov/gene/). Our up- and down-regulated gene lists (respectively composed of n = 133 and n = 255 Affymetrix probesets) yielded 117 and 220 comparable gene symbols. The final gene lists obtained and compared are presented in Additional file 2 and summarized descriptions of the individual studies, their sources of material, sample numbers, and microarray platforms used, are given in Table 3. Lists of genes in common between two or more studies were also submitted to DAVID for GO enrichment analysis. Finally, GSEA was used to determine the global distribution of lists of regulated genes within our ranked data set.

**Table 3:** Summary of independent microarray experiments compared in the present study

**Study**	**Material**	**Genotype/*n***	**Platform**
Clarke (this paper)	Native nasal epithelium (brushings)	5 CF (F508del homoz.) *vs.* 5 controls	Affymetrix Custom HsAirwaya520108F
Virella-Lowell *et al.*, 2004 [16]^1^	Isogenic bronchial cells (IB3-1 and S9)	F508del/W1282X vs. WT-CFTR corrected: 3 technical replicates each	Affymetrix U95Av2
Zabner *et al.*, 2005 [17]^1^	Primary tracheal and bronchial cell cultures	10 CF (F508del homoz.) *v*s. 10 controls	Affymetrix HGU-133A
Wright *et al.*, 2006 [18]^1,3^	Native nasal epithelium (brushings)	4 CF (F508del homoz.) vs. 12 controls	Affymetrix HGU-133A,B
Verhaeghe *et al.*, 2007 [20]^1^	Fetal tracheal cells (CFT-2 and NT-1)	F508del homoz. *vs*. WT-CFTR: 3 technical replicates each	Affymetrix HGU-133Plus2
Ogilvie et al., 2011 [19]^2^	Native bronchial (and nasal) epithelium (brushings)	F508del homoz. (in most cases) *vs*. controls: (8 vs. 15 bronchial; 20 vs. 16 nasal)	Illumina HumanRef-8 v1 Expression BeadChips

The table highlights the diversity of experimental design, materials and microarray platforms used in the five independent studies used for comparison with our data. ^(1)^We used the genelists resulting from Hampton & Stanton’s 2010 [21] reanalysis of these four data sets for our comparison. ^(2)^For our preliminary comparison with this study we used the published genelist for bronchial samples only. For our secondary comparison we reanalysed the data from both bronchial and nasal samples. ^(3)^The gene list used here represents the “mild” CF samples from this study only.

### Reanalysis of published dataset

For a more in-depth comparison between our gene list and the *in vivo* data from Ogilvie et al. (2011) [19], we reanalyzed their data (raw files available at http://www.ebi.ac.uk/arrayexpress/experiments/E-MTAB-360). The Bioconductor Lumi package (http://www.bioconductor.org/packages/2.11/bioc/html/lumi.html) was used for quality control and normalization. Following removal of two outlier samples, data from 78 Illumina HumanRef-8 v1 Expression BeadChips representing 20 CF nasal cell samples, 16 control nasal cell samples, 8 CF bronchial cell samples and 15 control bronchial cell samples (with some samples represented by 2–3 technical replicates) was quantile normalized and the normalized values subjected to RP analysis (CF-vs-control nasal and CF-vs-control bronchial), followed by detection call filtering (present at p < 0.01 in more than 10% of samples compared). Extended genelists were chosen using variable cutoffs as in exploratory gene array analysis [21], resulting in lists of 616 up- and 303 down-regulated genes in nasal epithelium and 441 up and 510 down-regulated genes in bronchial epithelium, including 69% and 70% of the originally published bronchial gene lists [19] (for gene lists resulting from new analysis see Additional file 3). For comparison of our dataset with the reanalysis of Ogilvie et al. (2011) [19], we lowered our fold change cutoffs and thereby extended our lists to comparable length, while still maintaining a false positive cutoff of pfp < 0.05 (for extended gene list from this study see Additional file 4). The final reanalyzed gene lists collapsed to single gene symbols and used for comparison are shown in Additional file 5. Regulated genes identified as shared were submitted to DAVID for GO analysis and GeneMania (http://www.genemania.org/) was used to generate a gene association network.

## Results

### Gene list

**Figure 1:**
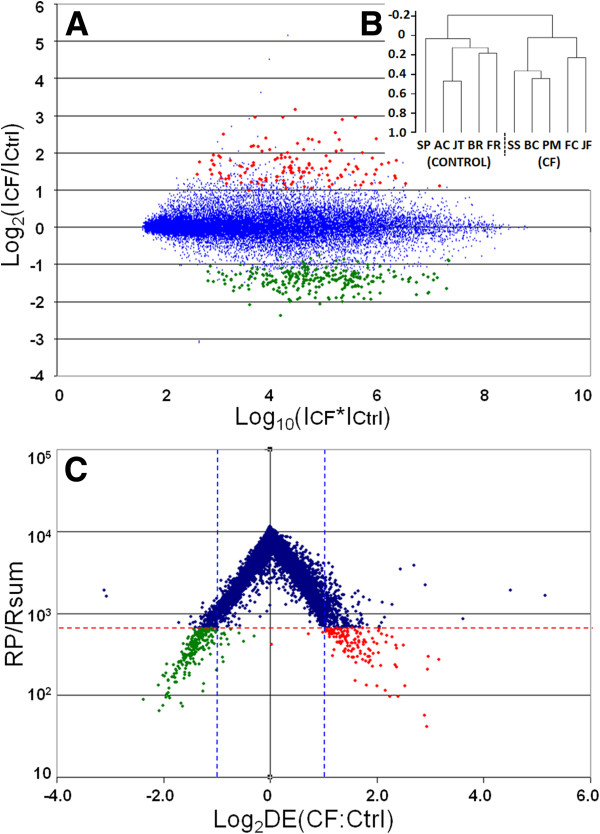
**Visualization of Microarray data. A**) R-I plot, in which the log_2_ of the ratio of mean intensities in CF-*vs.*-non CF samples is plotted against the log of their product, demonstrating the absence of an intensity dependent fold-change bias. The regulated genes chosen for further analysis using the RP statistic (p < 0.0001 cut-off) are shown in red (up-regulated genes) and green (down-regulated genes). **B**) Dendrogram of clustered samples, in which CF and non CF samples cluster with respect to their phenotype. **C**) Volcano plot of microarray data, in which the Log_2_ of the RP statistic is plotted against Log_2_ of Fold Change. The p < 0.0001 cut-off (horizontal dotted line) illustrates the gene list chosen for further analysis. Genes outside the vertical dotted lines have more than 2-fold differential expression.

Applying the Rank Products statistical package to the RMA-normalized data set resulted in 2 gene lists including all probesets present on the HsAirway microarray; one ranked with respect to probability of up-regulation in CF (termed “Up-CF”), and one ranked with respect to probability of down-regulation in CF (termed “Down-CF”). Irrespective of type I error (false positive probability/FDR) or p-value, there were 431 probesets with more than 2-fold up-regulation in the “Up-CF” list, and 420 probesets with more than 2-fold down-regulation in the “Down-CF” list. Applying cut-offs of FDR < 0.05 and p < 0.0001 to the RP-ranked gene lists (“Up-CF” and “Down-CF”) resulted in a list of 133 up-regulated probesets representing 117 named genes (123 transcripts for which a gene name was assignable, 6 of which were represented by two probesets, and 10 un-named probesets), and 255 down-regulated probesets representing 220 named genes (232 named genes, 10 of which were represented by two probesets, one of which was represented by three probesets, and 23 unnamed probesets). These strict gene lists are presented in Additional file 1. Figure 1A shows an R-I plot, in which the logarithm of the ratio of mean intensities in CF-vs-Control samples is plotted against the logarithm of their product, demonstrating the absence of an intensity dependent fold-change bias. The regulated genes chosen for further analysis using the Rank Products statistic (at the p < 0.0001 level) are highlighted in 2 different colours: red (up-regulated genes) and green (down-regulated genes). Figure 1B shows a dendrogram of clustered samples, in which CF and Control samples cluster with respect to their phenotype, as expected. Figure 1C shows a volcano plot in which the Log2 of the RP statistic is plotted against Log2 Fold Change. Introducing a p < 0.0001 cut-off illustrates the gene list chosen for further analysis and preliminary comparisons with other datasets, as described above, in which it can be noted that the vast majority of chosen genes have a fold change greater than 2 or less than 0.5.

### GO term enrichment and pathway analysis

The full list of 337 official gene symbols represented by the 388 regulated probesets was submitted to DAVID. Details of the resulting functional annotation are given in Table 4. The most enriched Biological Process (BP) GO term was found to be “negative regulation of cell proliferation” (p = 6.77E-04, n = 14 genes of which 12 are up-regulated in CF). The most enriched Cell Component (CC) GO term was “cilium” (p = 3.26E-05, n = 10 genes, all down-regulated in CF). Of the Molecular Function (MF) GO terms, the most enriched was “microtubule motor activity” (p = 0.00295, n = 6 genes, all down-regulated in CF), although the term “calcium ion binding” (p = 0.00298, n = 23 genes, 11 of which are up-regulated in CF) was also over-represented. DAVID also found strong tissue specific association of the gene list to lung (p = 6.08E-06, n = 63 genes, of which 37 are down-regulated in CF), and testis (p = 0.00259, n = 80 genes, of which 66 are down-regulated in CF).

**Table 4:** Functional enrichment from DAVID analysis

**Category**	**Term**	**Count**	**%**	**P**	**Fold Enrichment**	**FDR**
GOTERM_BP_FAT (Biological process)	GO:0008285 **negative regulation of cell proliferation**	14	5.1	6.77 E-04	3.1	1.12
	GO:0006953 **acute-phase response**	5	1.8	1.58 E-03	9.8	2.60
	GO:0008202 **steroid metabolic process**	9	3.3	4.16 E-03	3.5	6.70
	GO:0001960 **negative regulation of cytokine-mediated signaling pathway**	3	1.1	4.23 E-03	29.5	6.81
	GO:0042127 **regulation of cell proliferation**	20	7.3	5.03 E-03	2.0	8.04
	GO:0010038 **response to metal ion**	7	2.5	5.92 E-03	4.3	9.40
GOTERM_ CC_FAT (Cell compartment)	GO:0005929 **cilium**	10	3.6	3.26 E-05	6.2	0.04
	GO:0030286 **dynein complex**	6	2.2	5.81 E-05	14.1	0.07
	GO:0005615 **extracellular space**	22	8.0	1.16 E-04	2.6	0.15
	GO:0005930 **axoneme**	6	2.2	1.46 E-04	11.7	0.19
	GO:0044421 **extracellular region part**	26	9.5	3.26 E-04	2.2	0.42
	GO:0042995 **cell projection**	21	7.6	4.05 E-04	2.4	0.52
	GO:0005856 **cytoskeleton**	31	11.3	1.63 E-03	1.8	2.08
	GO:0005875 **microtubule associated complex**	7	2.5	1.70 E-03	5.5	2.16
	GO:0044430 **cytoskeletal part**	23	8.4	3.48E-03	1.9	4.39
	GO:0035085 **cilium axoneme**	4	1.5	3.55 E-03	12.8	4.48
	GO:0005576 **extracellular region**	39	14.2	4.51 E-03	1.6	5.65
GOTERM_MF_FAT (Molecular Function)	GO:0003777 **microtubule motor activity**	6	2.2	2.95 E-03	6.1	4.03
	GO:0005509 **calcium ion binding**	23	8.4	2.98 E-03	2.0	4.07
UP_TISSUE	**Lung**	63	22.9	6.08 E-06	1.8	0.01
	**Testis**	80	29.1	2.59 E-03	1.3	3.06
	**Trachea**	13	4.7	7.16 E-03	2.4	8.26
	**Plasma**	11	4.0	7.45 E-03	2.7	8.58

The most enriched GO terms found following submission of our gene list to the DAVID functional annotation tool, showing categories and GO terms enriched, number and percentage of gene lists genes represented in each enriched category, significance level (P), fold enrichment and false discovery rate (FDR).

The full list of 388 regulated probesets was also submitted to the GeneGo Metacore tool (http://thomsonreuters.com/products_services/science/systems-biology/), which comprises an integrated knowledge database and software suite for pathway analysis of gene lists. As well as providing a curated literature-based pathway analysis tool, Metacore generates lists of GO terms (Processes, Molecular Functions or Localizations) found to be enriched in the submitted gene list, which were similar to those obtained using DAVID. The 3 most enriched “GO Processes” were “acute phase response” (p = 1.19E-05, n = 7 genes), “response to glucocorticoid stimulus” (p = 1.86E-05, n = 12 genes) and “cell projection organization” (p = 3.92E-05, n = 21 genes). The 3 most enriched “GO Localizations” were “microtubule” (p = 4.12E-06, n = 15 genes), “microtubule cytoskeleton” (p = 5.50E-06, n = 22 genes) and “cilium” (p = 2.46E-05, n = 10 genes). Of the “GO Molecular Functions”, the most enriched was “calcium ion binding” (p = 1.35E-04, n = 26 genes).

Using Metacore to construct networks based on the best documented relationships between proteins encoded by list genes, there were found to be few direct interactions between proteins encoded by list genes, and only a limited number of known CFTR interacting proteins (namely up-regulated channel proteins CLCA2 and AQP9). Metacore found the gene list to be enriched in targets for several important transcription factors, including SP1 (45 genes, p = 3.64E-113), c-MYC (21 genes, p = 5.75E-52), ESR1 (20 genes, p = 1.86E-49) and NF-κB (20 genes, p = 1.86E-49). NF-κB has been implicated as a mediator of IL-8 inflammatory signalling in CF [39], but (along with SP-1 and c-MYC) NF-κB itself was not found to be regulated in our data set, although significant enrichment of the NF-κB pathway was detected by GSEA (see below).

**Figure 2:**
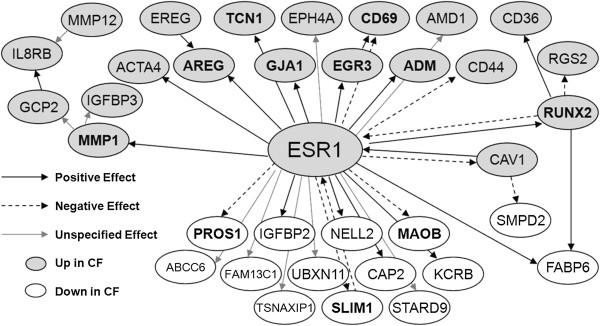
**ESR1 transcription targets regulated in CF.** Network adapted from Genego Metacore. Grey lozenges indicate genes up-regulated in CF (including ESR1); white lozenges indicate genes down-regulated in CF. Arrows represent effect predicted by Metacore of ESR1 on gene, as shown on key. Gene names shown in bold are those whose direction of regulation in CF would be reinforced by ESR1, based on its predicted effect.

The most interesting transcription factor identified in the context of our data was ESR1 (estrogen receptor 1), given a recent study of sex related differences in modulation of CF symptoms by estrogen [40], and the gene for ESR1, while not within the cut-offs of our working list (Additional file 1) was nevertheless 1.84-Fold up-regulated in CF (FDR = 0.026, p = 0.0004: see extended list in Additional file 4). ESR1 target genes in our gene list are shown in Figure 2, an adapted Metacore-generated network showing all detected direct interactions among list genes and ESR1.

### Relative qRT-PCR validation of microarray data

**Figure 3:**
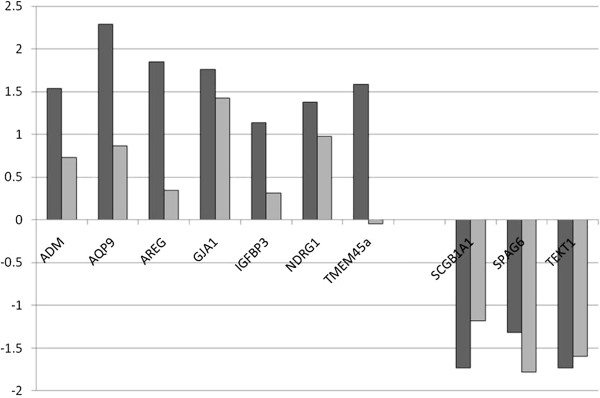
**Comparison of Microarray and qRT-PCR expression data for selected genes.** Comparison of Log_2_ (fold change) of gene expression in CF vs. Control nasal cell samples as measured by microarray analysis (black) and qRT-PCR (grey) using independent samples (n = 6 CF and 5 Ctrl in each case). Microarray data are log_2_(fold change) of normalized intensities, and qRT-PCR data are log_2_(mean fold change). Direction of differential expression was in agreement for all genes except TMEM45a, whose up-regulation in CF was not supported.

For validation of microarray data, we selected 10 genes of interest found either to be upregulated (n = 7) or downregulated (n = 3) in our CF microarray data (see Table 5). Comparison of fold-change of expression in CF nasal cells as measured by microarray analysis and qRT-PCR validation using independent tissue samples is shown in Table 5 and Figure 3. The direction of differential expression measured was in agreement for 6/7 upregulated genes (ADM, AQP9, AREG, GJA1, IGFBP3 and NDRG1) and 3/3 downregulated genes (SPAG6, TEKT1 and SCGB1A1), although by two methods statistical analyses of mean dCTs (Student’s t test and Wilcoxon rank sum) did not reach significance due to the low number of replicates (t test values shown in Table 5).

**Table 5:** Genes for which differential expression was reanalysed by qRT-PCR in independent nasal cell samples

**Gene symbol**	**Affymetrix ID**	**Gene description**	**FC (Array)**	**FC (QPCR)**	**p (t test)**
*Upregulated in CF*					
**ADM**	202912_at	Adrenomedullin	2.9	1.7	0.08
**AQP9**	205568_at	Aquaporin 9	4.9	1.8	0.41
**AREG**	205239_at	Amphiregulin	3.6	1.3	0.21
**GJA1**	201667_at	Gap junction protein alpha 1 or Connexin 43	3.4	2.7	0.22
**IGFBP3**	210095_s_at	Insulin-like growth factor binding protein 3	2.2	1.2	0.49
**NDRG1**	200632_s_at	N-myc downstream regulated gene 1	2.6	2.0	0.18
**TMEM45a**	219410_at	Transmembrane protein 45A or DERP7	3.0	1.0	0.5
*Downregulated in CF*					
**SCGB1A1**	205725_at	Secretoglobin family 1A member 1 or Uteroglobin	−3.3	−2.3	0.11
**SPAG6**	210033_s_at	Sperm associated antigen 6	−2.5	−3.4	0.1
**TEKT1**	239216_at	Tektin 1	−3.3	−3.0	0.12
*reference genes*					
**ACTB**	*3 probesets*	Actin beta	*mean 1.2*	*1.2*	/
**GAPDH**	*3 probesets*	Glyceraldehyde 3-phosphate dehydrogenase	*mean 1.1*	*0.8*	/

Table shows gene symbols and description, Affymetrix probeset ID as represented on the HsAirway array, fold change in CF-*vs.*-non CF samples for both microarray and qRT-PCR, and p value for qRT-PCR data.

### Comparison with other studies

**Figure 4:**
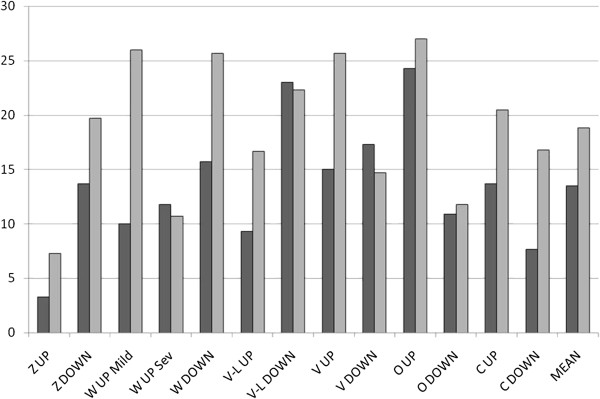
**Histogram showing percentages of genes in common among lists of differentially expressed genes from six microarray studies of CF related gene expression.** Percentages are of number of regulated genes in one study regulated in the same direction (dark columns) or showing inverted expression (light columns) in the other five studies (Z: Zabner et al., 2005; W: Wright et al., 2006; V-L: Virella-Lowell et al., 2004; V: Verhaeghe et al., 2007; O: Ogilvie et al., 2011; C: current study). MEAN refers to mean percentage of all columns (data are summaries of percentages shown in Table 6).

The percentage of genes shared between lists of up- and down-regulated genes from 5 other studies [16-20] and the present study are shown in Table 6 and summarized in Figure 4. Although every study has some up- and down-regulated genes in common with every other (ie, with the same direction of differential expression in both studies), it is interesting to note that the majority of comparisons yield similar numbers of genes with inverted direction of expression (on average, 18% of list genes from each study appeared at the opposite end of at least one other study) to genes differentially expressed in the same direction (mean = 13% of genes from one study appearing at same end of at least one other study, see Figure 4).

**Table 6:** Percentages of genes in common among differentially expressed genes from six microarray studies of CF related gene expression

***% shared***	***Z***	***W***	***V-L***	***V***	***O***	***C***
**UP**	(N = 300)	(N = 300)	(N = 300)	(N = 300)	(N = 115)	(N = 117)
**Z**	*-*	0.7	0.3	1.0	1.7	1.7
**W**	0.7	*-*	3.7	5.0	2.6	1.7
**V-L**	0.3	3.7	*-*	4.0	2.6	1.7
**V**	1.0	5.0	4.0	*-*	14.8	4.3
**O**	0.7	1.0	1.0	5.7	*-*	7.7
**C**	0.7	0.7	0.7	1.7	7.8	*-*
**ALL**	**3.3**	**10.0**	**9.3**	**15.0**	**24.3**	**13.7**
**DOWN**	(N = 300)	(N = 300)	(N = 300)	(N = 300)	(N = 110)	(N = 220)
**Z**	*-*	6.7	5.3	3.3	2.7	0.5
**W**	6.7	*-*	7.0	2.7	1.8	1.4
**V-L**	5.3	7.0	*-*	10.3	2.7	2.3
**V**	3.3	2.7	10.3	*-*	0.9	3.2
**O**	1.0	0.7	1.0	0.3	*-*	1.4
**C**	0.3	1.0	1.7	2.3	2.7	*-*
**ALL**	**13.7**	**15.7**	**23.0**	**17.3**	**10.9**	**7.7**
***% inverted***	***Z***	***W***	***V-L***	***V***	***O***	***C***
**UP**	(N = 300)	(N = 300)	(N = 300)	(N = 300)	(N = 115)	(N = 117)
**Z**	*-*	1.3	1	4.3	1.8	0.9
**W**	7	*-*	6	5.7	6.4	12.7
**V-L**	7.7	3.7	*-*	4.7	1.8	1.4
**V**	3.7	12.3	13	*-*	0	1.4
**O**	1	7	3.3	0	*-*	1.8
**C**	1.3	3.7	1.7	1.3	2.7	*-*
**ALL**	**19.7**	**25.7**	**22.3**	**14.7**	**11.8**	**16.8**
**DOWN**	(N = 300)	(N = 300)	(N = 300)	(N = 300)	(N = 110)	(N = 220)
**Z**	*-*	7	7.7	3.7	2.6	3.4
**W**	1.3	*-*	3.7	12.3	18.3	9.4
**V-L**	1	6	*-*	13	8.7	4.3
**V**	4.3	5.7	4.7	*-*	0	3.4
**O**	0.7	2.3	0.7	0	*-*	2.6
**C**	0.7	9.3	1	1	3.5	*-*
**ALL**	**7.3**	**26**	**16.7**	**25.7**	**27.0**	**20.5**

Percentages are of number of regulated genes in study indicated in horizontal row regulated in the same direction or showing inverted expression in the other five studies as shown at left (Z: Zabner et al., 2005 [17]; W: Wright et al., 2006 [18]; V-L: Virella-Lowell et al., 2004 [16]; V: Verhaeghe et al., 2007 [20]; O: Ogilvie et al., 2011 [19]; C: current study). In each case, ALL refers to total percentage of genes shared or inverted between one study and all five other studies (summarized in Figure 4).

The identity of the 75 up-regulated and 114 down-regulated genes shared (and sharing direction of differential expression) between two or three of the compared studies (this study plus [16-20]) is given in Table 7. This shared gene list was submitted to DAVID to detect enrichment of GO terms, and the results are summarized in Table 8. “Regulation of cell proliferation” (15 genes; p = 2.48E-05) and “immune response” (16 genes; p = 2.20E-04) were the most enriched biological process (BP) GO terms among shared up-regulated and down-regulated genes respectively, and overall “Defence Response” (25 genes; p = 6.00E-07) was the most enriched BP GO term among all shared genes. The top cell compartment (CC) term was “extracellular region” for both up and down-regulated shared genes (49 genes for combined list; p = 1.88E-07), and the top molecular function (MF) GO terms were “enzyme binding” (up: 8 genes, p = 0.0092) and “MHC class II receptor activity” (down: 4 genes, p = 3.17E-04), with enzyme inhibitor activity being the most enriched MF GO group for all regulated genes (13 genes, p = 7.39E-05). Not surprisingly, “Lung” was the most enriched UP-Tissue class (52 genes, p = 5.15E-07), with 34 (65%) of the genes identified as belonging to this class being down-regulated in CF.

**Table 7:** Differentially expressed genes common to two or more of six comparable studies of CF related gene expression

**Gene list**	**Regulated genes shared with current study (Clarke)**
Zabner et al., 2005	**UP:** ACAA2, CDKN2B.
	**DOWN:**IGFBP2.
Wright et al., 2006	**UP:** C9orf3, KRT14.
	**DOWN:**CYP24A1, HLA-DQA1, SAA4.
Virella-Lowell et al., 2004	**UP:** CAV1, CCNE2.
	**DOWN:** CLGN, ENO2, EPB41L3, GPX3, TIMP4.
Verhaeghe et al., 2007	**UP:**BCL2A1, G0S2, IL1B, MMP1, RGS2.
	**DOWN:** CKB, CRIP1, CYP24A1, DNALI1, FHL1, GSTT1, IGFBP2.
Ogilvie et al., 2011	**UP:**BCL2A1, G0S2, IL1B, IL1R2, LCP2, NDRG1, RGS2, RNF149, TCN1.
	**DOWN:** PROS1, SCGB1A1, SPAG8.
**All shared genes (six studies combined)**	
**UP **(n = 75): *ACAA2*, AGL, AKR1C1, ANXA8L2, BCHE, BLOC1S1, BTBD3, *C9ORF3*, CAPG, *CAV1*, CCL20, *CCNE2*, CD24, CD83, *CDKN2B*, CLGN, CSF3, DDX3Y, ELK3, FOLR1, FOLR3, FOXG1, GCA, GFPT2, HCLS1, HIST1H1C, HMOX1, HPCAL1, HSPB11, IFIT1, IFIT3, IFITM1, *IL1R2*, IL7R, ISG15, KCTD12, *KRT14*, KRT81, *LCP2*, LITAF, LYPD1, MLF1, *MMP1*, MRPL28, MX2, NCF1, *NDRG1*, NET1, PLAU, PLAUR, PLTP, PRSS3, PSG9, PTPN13, RAC2, RAGE, *RNF149*, RPA3, SEMA3B, SERPINA3, SERPINF1, SLITRK5, SOD2, SULT1A3, TCF15, TCIRG1, *TCN1*, TXNIP, *BCL2A1*, *G0S2*, *IL1B*, NCF2, PLAT, PTGS2, *RGS2*.	
**DOWN** (n = 114): ACTA2, ADAR, ALDH1A1, ANKRD1, ASNS, ASS1, BEX4, BST2, BTG1, C5ORF13, CALD1, CAP1, CCL20, CD164, CFB, CGREF1, *CKB*, *CLGN*, COL8A1, COL9A3, *CRIP1*, CSTA, CXCR4, CYP51A1, DDB2, DDIT4, *DNALI1*, DSP, DSTN, DYNLT1, EDNRA, EFEMP1, EIF4A2, *ENO2*, *EPB41L3*, EPS8, F3, FBLN5, FCGBP, *FHL1*, GABRP, GCH1, GINS1, GPNMB, GPR1, *GPX3*, *GSTT1*, GZMB, HCP5, HES1, HLA-B, *HLA-DQA1*, HLA-DRA, HLA-DRB1, HLA-F, HLA-G, HMGCS1, HSPB1, HTRA1, ID1, IFI16, IFITM1, IFRD1, IGFBP7, IL32, KCNN4, KIT, KLRK1, KRT15, LCN2, LGALS3BP, LOX, MGAM, MSC, NID2, NPR3, NTS, PGD, PNMA2, PPP1R3C, *PROS1*, RASGRP1, RND3, RUNX3, *SAA4*, SC5DL, *SCGB1A1*, SERPINB3, SERPINB4, SGK1, SLC2A3P1, SNAPC1, *SPAG8*, STAC, STAT4, TES, TFPI, THBD, *TIMP4*, TPBG, TRIB2, TWIST1, VDAC1, ZNF643, CTSC, *CYP24A1*, IFI27, *IGFBP2*, IGFBP3, NAMPT, PRSS23, SEL1L3, TMSB4X, TRIM22.	

Genes shared between two other lists and the present study (ie, differentially expressed in the same direction with respect to CF-vs.-Control cells) are underlined. The lower panel shows genes shared between two or more studies when all six studies are combined. Genes shared with present study (see upper panel) are shown in italics, genes shared between three studies are underlined.

**Table 8:** Functional enrichment analysis of genes shared between two or three studies

**Category**	**Term**	**Count**	**%**	**p**	**Fold enrichment**	**FDR**
GOTERM_BP_FAT	GO:0006952 **defence response**	25	13.4	6.00E-07	3.25	0.001
	GO:0042060 **wound healing**	14	7.5	7.37E-07	5.87	0.001
	GO:0042127 **regulation of cell proliferation**	28	15.1	1.37E-06	2.85	0.002
	GO:0006955 **immune response**	25	13.4	4.51E-06	2.90	0.008
	GO:0006928 **cell motion**	20	10.8	6.96E-06	3.37	0.012
GOTERM_CC_FAT	GO:0005576 **extracellular region**	49	26.3	1.88E-07	2.12	0.000
	GO:0044421 **extracellular region part**	29	15.6	3.41E-06	2.63	0.004
	GO:0005615 **extracellular space**	23	12.4	1.01E-05	2.92	0.013
	GO:0000267 **cell fraction**	27	14.5	2.17E-04	2.17	0.275
	GO:0005625 **soluble fraction**	13	7.0	2.48E-04	3.61	0.314
GOTERM_MF_FAT	GO:0004857 **enzyme inhibitor activity**	13	7.0	7.39E-05	4.11	0.101
	GO:0032395 **MHC class II receptor activity**	4	2.2	0.001303	17.98	1.768
	GO:0004866 **endopeptidase inhibitor activity**	8	4.3	0.001519	4.71	2.058
	GO:0030414 **peptidase inhibitor activity**	8	4.3	0.002074	4.47	2.800
	GO:0005520 **insulin-like growth factor binding**	4	2.2	0.00294	13.67	3.947
KEGG_PATHWAY	hsa05332: **Graft-versus-host disease**	8	4.3	2.41E-06	12.42	0.003
	hsa04940: **Type I diabetes mellitus**	8	4.3	4.07E-06	11.53	0.004
	hsa05330: **Allograft rejection**	7	3.8	2.08E-05	11.77	0.023
	hsa04610: **Complement and coagulation cascades**	8	4.3	1.14E-04	7.02	0.126
	hsa05416: **Viral myocarditis**	8	4.3	1.37E-04	6.82	0.151
UP_TISSUE	**Lung**	52	28.0	5.15E-07	2.03	0.001

The most enriched GO terms found by the DAVID functional annotation tool among the 75 up-regulated and 114 down-regulated genes shared between two or three of the 6 compared studies (see Table 7), showing categories and GO terms enriched, number and percentage of gene lists genes represented in each enriched category, p value, fold enrichment and false discovery rate (FDR).

### Gene set enrichment analysis

**Figure 5:**
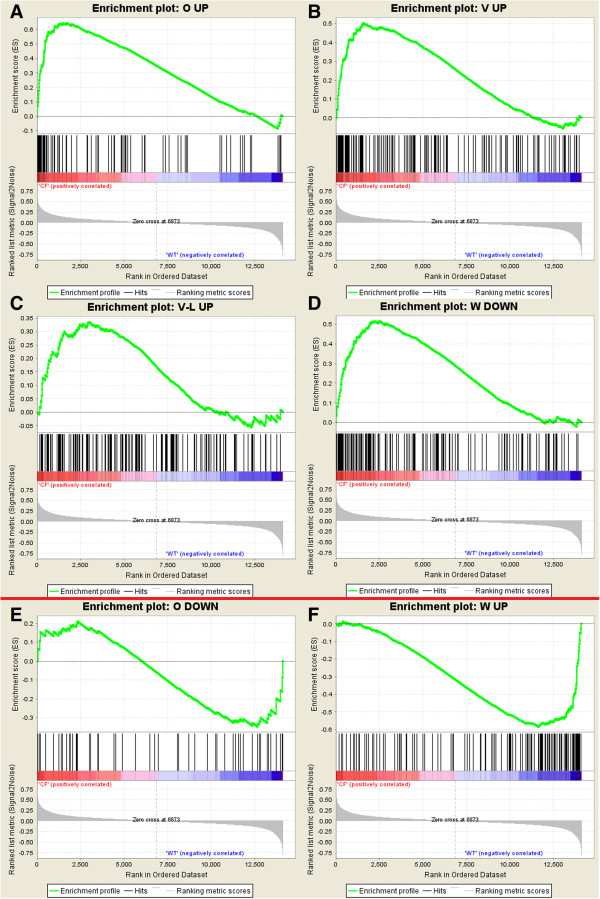
**Comparison of independent microarray studies and present study by GSEA.** GSEA enrichment plots showing non-significant enrichment of up-regulated genes from **A**) Ogilvie et al. (2011), **B**) Verhaeghe et al. (2007), **C**) Virella-Lowell et al. (2004), and down-regulated genes from **D**) Wright et al. (2006) at the CF end of our dataset (red bar), and non-significant enrichment of down-regulated genes from **E**) Ogilvie et al. (2011), and up-regulated genes from **F**) Wright et al. (2006), at the control end of our dataset (green bar), demonstrating a partial inversion of CF-related gene expression between our study and Wright et al. (2006).

The Gene Set Enrichment Analysis (GSEA) software package was used to localize the differentially expressed genes from each of five other studies [16-20] in our ranked data set. The results of this analysis (see Figure 5), although not significant, show agreement among genes up-regulated in the F508del-CFTR condition between our study and three others [16,19,20], and some enrichment of down-regulated genes from Ogilvie et al. [19] at the down-regulated end of our ranked list. Inversion of both up and down-regulated genes from another nasal cell study [18] (“CF-mild” samples only) was also demonstrated. As the Wright et al. [18] data set we used in comparison omitted samples classified as CF-severe [21], we wondered if this inversion of data sets might result from the use of only CF-mild samples. We therefore repeated the analysis using the published list of genes found to be up-regulated in CF-severe samples in that study [18] (n = 592 genes) in GSEA analysis of our data set, and found that as for the CF-mild samples, a proportion (18%) of the genes up-regulated in severe CF samples in that study were present at the control end of our data set (ie, down-regulated in CF), confirming that a proportion of the data is inverted between these two studies.

**Figure 6:**
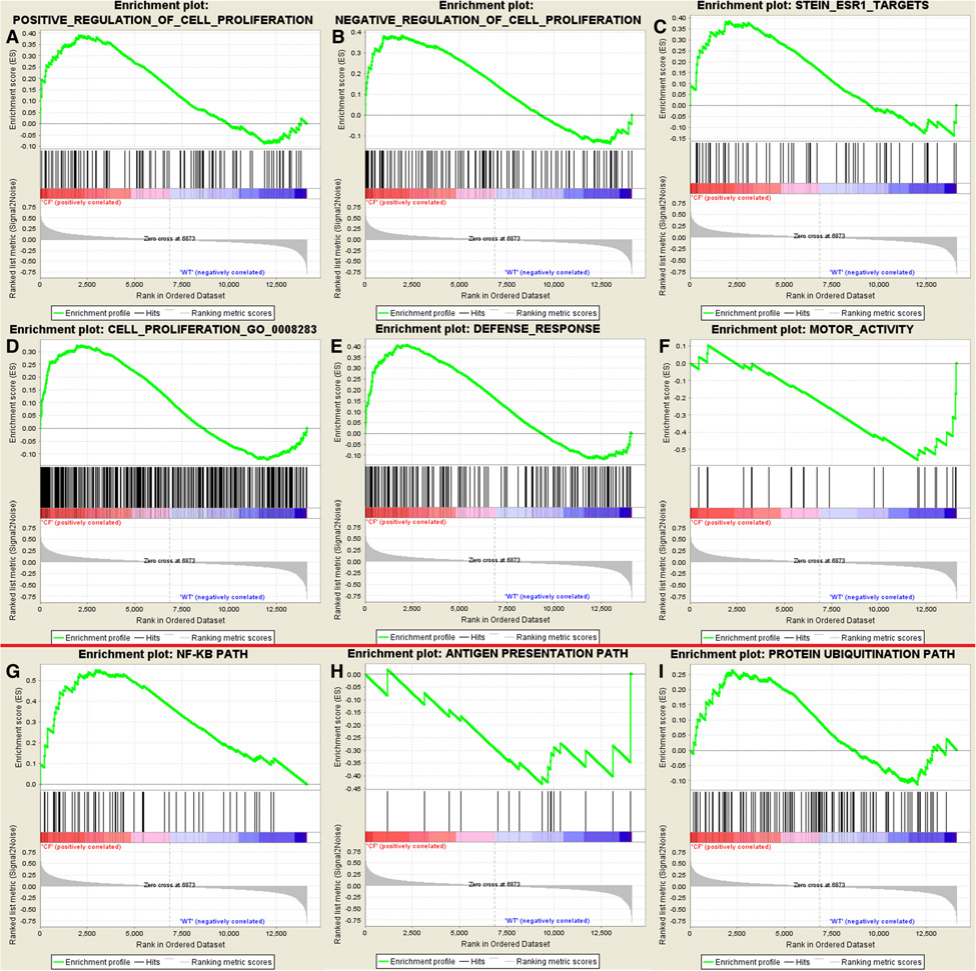
**Demonstration of pathway enrichment in present study by GSEA.** GSEA enrichment plots showing significant enrichment (ES significant at p < 0.05 level) of genesets for **A**) positive and **B**) negative regulation of cell proliferation, **C**) ESR1 targets, **D**) cell proliferation and **E**) defence response for the CF phenotype, and **F**) motor activity for the non CF phenotype. The lower panel shows **G**) significant enrichment of the NF-kB pathway in the CF phenotype and non-significant skews of **H**) antigen presentation pathway genes in the non CF phenotype and **I**) protein ubiquitination genes in the CF phenotype.

Further GSEA analyses were performed with gene sets relevant to the GO terms found to be enriched in our data set and in other CF related microarray studies. Examples where the enrichment is significant are presented in Figure 6. Concordant with our DAVID analysis, gene sets related to cell proliferation, and both positive and negative regulation thereof, were significantly enriched at the up-regulated end of our ranked data set, as were gene sets for ESR1 targets and defence response genes. Gene sets for genes involved in cytokine activity and Ca^2+^ binding showed borderline enrichment for the CF phenotype. At the down-regulated end of our ranked gene list (WT phenotype) we observed significant enrichment of motor activity gene expression, but only borderline enrichment of cell projection and testis gene sets. We also tested the three pathways (NF-κB, Antigen presentation and Protein ubiquitination) highlighted in a previous meta-analysis of CF microarray data sets [21] and found significant enrichment in the CF phenotype of genes in the NF-κB pathway (see Figure 6). The protein ubiquitination pathway showed borderline non-significant enrichment for the CF phenotype, and the antigen presentation pathway, although showing some enrichment for the down-regulated end of our data set, was also not significant.

### Reanalysis of independent dataset, and comparison with our data

**Figure 7:**
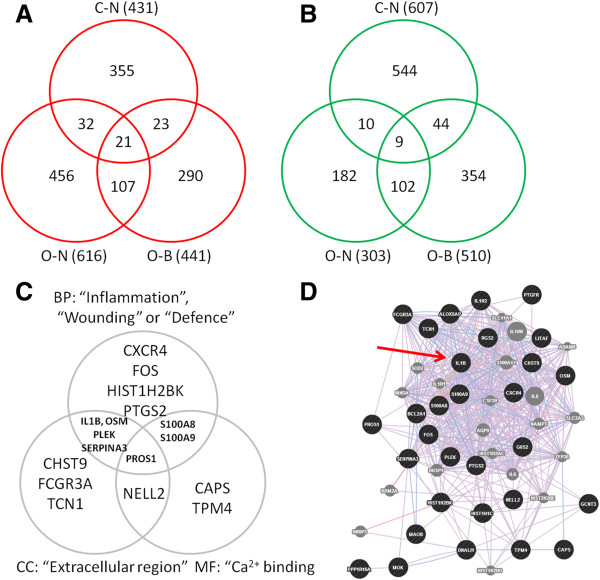
**CF gene signature in native airway tissues.** Representations of a putative gene signature in native airway tissues. **A**) and **B**) Venn diagrams showing numbers of up-regulated (**A**), and down-regulated (**B**) genes shared between the present study (C-N: Clarke nasal) and the reanalyzed study [19] (O-N: Ogilvie nasal; O-B: Ogilvie bronchial [19] in F508del-CFTR expressing vs. control airway epithelial cells. Numbers in brackets refer to total number of genes in each list, and numbers at centre of each diagram refer to the 30 genes shared between all three lists (see Table 9). **C**) Venn diagram distribution of shared genes among the most significant GO terms for biological process (BP), cell compartment (CC) and molecular function (MF) as revealed by DAVID analysis. **D**) GeneMania network showing relationships between 30 shared genes (black circles) and other connecting genes (grey circles). Relationships are divided into co-expression (purple lines), co-localization (blue lines), pathway (light blue lines), physical interactions (pink lines) and predicted (orange lines). The red arrow indicates IL1B, which is the most connected gene involved with the highest number of identifiable functions including inflammation, defence, response to bacteria, and cytokine production.

The results of our RP reanalysis of CF related gene expression in native nasal and bronchial cell samples [19] are shown in Additional file 3, and collapsed lists taking multiple probes into account for comparison presented in Additional file 5 alongside our gene lists, which were expanded to similar lengths for comparative purposes by relaxation of the cut-offs (see Additional file 4). A Venn diagram showing up- and down-regulated genes shared between our study and the reanalysed Ogilvie et al. [19] study is shown in Figure 7. Interestingly, the data reveal a marked agreement between both up and down regulated genes in nasal and bronchial cells from the reanalysed study, and 21 up-regulated and 9 down-regulated genes were shared by our study and both tissues in the reanalysed study (see Table 9). Submission of the list of 30 shared genes to DAVID revealed significant enrichment for the biological process GO terms “inflammatory response” (p = 4.8E-4), “response to wounding” (p = 7.9E-5) and “defence response” (p = 1.4E-3), the cell compartment term “extracellular region” (p = 2.0E-2) and the molecular function term “calcium ion binding” (p = 3.3E-2). The distribution of the genes between these GO terms is shown in Figure 7C. An association network was generated using the GeneMania program (http://www.genemania.org/): the 30 shared genes are shown as nodes in an association network in which the edges are coloured according to the category of interaction (see Figure 7D). Other genes relevant to CF pathology (eg, IL8) are shown as important connectors in this network. The list gene with the most interactions in this network was IL1B, which was also associated by GeneMania with the most known functions in relation to other members in the network, including inflammatory and defence responses, cytokine production, signaling regulation and proliferation.

**Table 9:** Small molecular signature for native CF airway epithelial cells

**Upregulated**	
**ALOX5AP**	arachidonate 5-lipoxygenase-activating protein; #241
**BCL2A1**	BCL2-related protein A1; #597
**CXCR4**	chemokine (C-X-C motif) receptor 4; #7852
**FCGR3A**	Fc fragment of IgG, low affinity IIIa, receptor (CD16a); #2214
**FOS**	FBJ murine osteosarcoma viral oncogene homolog; #2353
**G0S2**	G0/G1switch 2; #50486
**GCNT3**	glucosaminyl (N-acetyl) transferase 3, mucin type; #9245
**HIST1H1C**	histone cluster 1, H1c; #3006
**HIST1H2BK**	histone cluster 1, H2bk; #85236
**IL1B**	interleukin 1, beta; #3553
**IL1R2**	interleukin 1 receptor, type II; #7850
**LITAF**	lipopolysaccharide-induced TNF factor; #9516
**OSM**	oncostatin M; #5008
**PLEK**	pleckstrin; #5341
**PTGS2**	prostaglandin-endoperoxide synthase 2; #5743
**RGS2**	regulator of G-protein signaling 2, 24 kDa; #5997
**S100A8**	S100 calcium binding protein A8; #6279
**S100A9**	S100 calcium binding protein A9; #6280
**SERPINA3**	serpin peptidase inhibitor, clade A, member 3; #12
**TCN1**	transcobalamin I (vitamin B12 bp, R binder family); #6947
**TPM4**	tropomyosin 4; #7171
**Downregulated**	
**CAPS**	calcyphosine; #828
**CHST9**	carbohydrate (N-acet.gal.am. 4–0) sulfotransferase 9; #83539
**DNALI1**	dynein, axonemal, light intermediate chain 1; #7802
**MAOB**	monoamine oxidase B; #4129
**NELL2**	NEL-like 2 (chicken); #4753
**PPP1R16A**	protein phosphatase 1, regulatory subunit 16A; #84988
**PROS1**	protein S (alpha); #5627
**PTGFR**	prostaglandin F receptor (FP); #5737
**RAGE**	MOK protein kinase; #5891

Regulated genes shared between our study and reanalysed lists from another recent study [19]. Gene symbols, descriptions and Entrez IDs (#) are shown.

## Discussion

The present study of global gene expression in nasal epithelial cell samples from CF patients and healthy controls yields a snapshot of the CF transcriptome providing interesting insights into the consequences of CFTR dysfunction. Our primary aim was the identification of a CF molecular signature – a robust set of genes with potential utility as diagnostic markers or as targets for future therapeutic strategies. The approach we used – applying the same statistical method to both newer and older data, allowed us to propose such a signature, while also shedding light on the limitations of such snapshot studies in measuring a system as subtle and dynamic as the transcriptome.

### Our data

Many of the individual genes within our microarray gene list are of known functional significance in CF pathophysiology, but for a better understanding of the cellular processes and pathways altered in CF epithelium we utilised Gene Ontology (GO) term enrichment in the whole lists of 133 up-regulated and 255 down-regulated genes. The most highly enriched GO terms were “negative regulation of cell proliferation” (biological process), mainly composed of up-regulated genes, “cilium” (cell compartment), and “microtubule motor activity” (molecular function) which were both composed of only down-regulated genes. “Extracellular space” (cell compartment) and “calcium ion binding” (molecular function) also accounted for a significant number of regulated genes in both directions (see Table 4). Taken together, these systemic alterations of gene expression might imply that CFTR dysfunction causes: 1) ER stress and alteration of calcium signalling, plausibly to activate alternative chloride channels, 2) disturbances in the normal processes of epithelial cell differentiation and extracellular signalling, and 3) a reduction in ciliogenesis or cilia activity.

#### Proliferation and inflammation

In airway epithelium, the proliferating cell population is likely to be composed of the basal-like, rather than the ciliated epithelial cells [41], and in fact the two populations can be seen as extremes of a proliferation-differentiation continuum. Of the 14 genes classified as negative regulators of cell proliferation (anti-proliferative) GO group, 12 (86%) were up-regulated in CF, suggesting that, overall, proliferation is reduced in CF. However, GSEA analysis showed significant enrichment of proliferative genes in general, and sets of both positive and negative regulators of proliferation in CF (see Figure 6). In similar situations elucidation of the function of each individual regulated protein builds up a picture of a complex network of apparently contradictory processes. Examples found here to be up-regulated in CF include ADM (adrenomedullin), a vasodilator which promotes alveolar development and repair [42], and which is speculated to have a protective effect in the immuno-inflammatory process of asthma [43], implying that it may respond to airway injury in CF. EREG (epiregulin), also up-regulated in CF, is a member of the epidermal growth factor (EGF) family generally associated with enhanced proliferation, but which in ciliated human airway epithelial cells can act via ERBB2 binding to maintain their differentiated phenotype [44]. GJA1 (Connexin 43), also up-regulated in CF, suppresses cell proliferation via maintenance of cell-cell communication, possibly via an association with CAV1 [45], which has an important role in maintenance of airway ECM integrity via inhibition of the TGF beta-induced fibrosis [46], and may also play an important role in modulating the immune response to *P. aeruginosa* infection through the formation of CFTR-expressing lipid rafts [47]. Other genes in this group are inflammatory cytokines (IL1B) or potentiate cell proliferation (FGFBP1, IGFBP3), and upon inspection there are few *bona fide* inhibitors of proliferation (OSM, CDKN2B). Taken together, the up-regulated genes belonging to the “negative regulation of Cell Proliferation” GO group may therefore represent a complex reaction to the effects of hyper-inflammation and tissue injury which are hallmarks of the CF airway. Given, however, that this was measured in the nasal epithelium, which is free of much of the pathology inherent in the lower CF airway, it can also be argued that these patterns, including the hyperinflammatory component, form part of a primary response to CFTR dysfunction. It is nevertheless probable that while some of these genes are deregulated as a direct consequence of absence of CFTR, others are secondary, i.e., actively involved in balancing the negative effects of the former.

AREG (amphiregulin), identified here as up-regulated in CF, is a binding partner of EREG and a ligand of EGFR which was a representative of “extracellular space”, another highly enriched GO group. AREG is present in the sputum of CF patients [48], and is involved in both proliferation and inflammation in human airways [49,50]. Its expression in CF airway and blood neutrophils [48], might be suggestive that a component of the gene expression observed in nasal cell samples analysed here was derived from the 5-10% of inflammatory cells present (see Table 1). However, neutrophil genes in general were not over-represented in our gene lists: only 4 genes stringently identified as regulated in CF airway neutrophils [48] were present among our up-regulated genes (CXCR2, CXCL9, CXCL10, and ADM, which is supposedly down-regulated in CF neutrophils [48]), and none among our down-regulated genes. Most of our CF gene expression profile can therefore be associated to the nasal epithelial cell population, and the presence on our list of several genes associated with inflammation lends support to the idea that CFTR dysfunction on its own can stimulate inflammatory signalling to some extent.

#### Cilium

The cilium is an organelle directly affected by CF pathophysiology, given its role in mucus clearance and the physical barrier to such clearance in CF. Given that the CF nasal epithelium is not burdened with abnormally thick mucus to the same extent as the CF lung, the down-regulation of cilia genes in this tissue suggests a primary disruption in CFTR related signalling rather than a secondary response related to abnormal mucus. Of the 10 down-regulated genes in this GO group, 5 are axonemal components (DNAH9, DNAH12, DNAI1, DNAI2 and DNAAF1/LRRC50) and 4 of the others are clearly involved in cilium or sperm flagellum function. Furthermore, other down-regulated genes which were not flagged in this GO term by DAVID can clearly be assigned to this group, increasing its significance (DNAH6, DNALI1, DNAAF3/ LOC352909 and TEKT1). Down-regulation in CF was broadly confirmed for two of these genes (SPAG6 and TEKT1) by rtQ-PCR in independent nasal cell samples (see Figure 3).

Suppression of cilia gene expression as a primary consequence of CFTR dysfunction might complicate the already compromised mucociliary clearance that is a hallmark of CF. Furthermore, many of these genes are also relevant to spermatogenesis or sperm motility via their role in the flagellum, and might therefore be relevant to cases of CF-associated male infertility (non-CBAVD [51-53]). Interestingly, GJA1 (CX43), an up-regulated gene in CF, has a functional link to cilia, given that in epithelial cells of nasal mucosa, only functional gap junctions of GJA1 are expressed [54], and it is through these junctions that the intracellular calcium wave that controls the beating of cilia is communicated. Taken together, our expression data suggest that CFTR dysfunction might predispose the airway to suboptimal cilia function, thereby compounding the CF phenotype.

#### Estrogen receptor targets

Several of our list genes are targets of the transcription factor ESR1 (see Figure 2), and targets for ESR1 were found to be significantly enriched in our CF samples by GSEA (see Figure 6). The presence of CF up/down-regulated genes in a network provides clues not only on how expression is affected by CFTR dysfunction, but also the opposite, e.g., how systemic alterations in circulating estrogen over the course of the female menstrual cycle, might bring about differential gene expression profiles, which help to explain subtle differences in lung function in male and female CF patients [40,55].

Our data on changes in gene expression in proliferation pathways, calcium, membrane and cilia biology can all be related back to the defect in CFTR processing, and can potentially be characterized within a model of a perturbed CFTR interactome [15]. The involvement of estrogenic signalling, however, introduces an “external modifier” providing a feasible explanation for some of the variability within a heterogeneous group of subjects. Although functional data are outside the scope of the present study, the ESR1 network identified here constitutes a source for clues as to how there may be crosstalk between different mechanisms during dysregulation of gene expression in CF. A sharp and sustained rise in circulating estrogen and its presumably positive effect on expression of several genes involved in regulating the transition between proliferative and differentiating cellular phenotypes (MMP1, ADM, AREG, GJA1, RUNX2: all found here to be up-regulated in CF, see Figure 2) might be a key factor in determining the equilibrium between these two states in the female CF airway epithelium.

### Comparison with other studies

The present study provides a momentary snapshot of Cystic Fibrosis-related gene expression in native nasal epithelial cell samples from CF patients compared to controls. We compared our gene-lists with those from other studies in an attempt to quantify the similarity between data sets. These comparative data enabled us to gauge which of the six studies are more alike in terms of the numbers of up-regulated and down-regulated genes they have in common (see Table 6), showing, for example, that our data have more up-regulated genes in common with a bronchial cell dataset [19] and more down-regulated genes in common with the study using immortalized foetal tracheal cells [20]. These results are set against a background of similar numbers of genes whose direction of expression is inverted between studies (see Figure 4). GSEA data point to partial inversion of gene regulation between our study and one also using native nasal epithelial cells [18] (see Figure 5D,F), but numbers of regulated genes also show inverted expression between several other studies (see Table 6). It is possible to speculate that the direction of CF marker gene regulation might not be as important as the fact of their deregulation in CF, and that the appearance of pathophysiologically relevant genes at different extremes of distinct studies might simply reflect fluctuations in what is a cyclical process of infection, inflammation, and airway repair, but in reality, the presence of biological replicates should cancel out any such effects. Three pathways previously suggested to be characteristic of CF-related gene expression [21], were found to be enriched to varying degrees in our data set (see Figure 6), including significant enrichment of the NF-kB pathway as previously noted in foetal tracheal cell lines [20], and used as evidence of intrinsic hyper-inflammation in CF. The fact that this is observed in nasal epithelium, along with over-expression of several genes related to the regulation of the inflammation does lend support to the presence of an intrinsic hyperinflammatory response associated with F508del-CFTR expression without, however, clarifying its origin [56]. The shared down-regulation of the antigen presentation pathway suggested by Hampton & Stanton [21] was also seen to some extent, and these data help to characterize our data set as belonging to the same group as the other CF data sets. Taken together (see Table 7) the 189 genes which share similar expression between 2 or 3 studies are enriched for functional categories (eg, defence response, wound healing, regulation of cell proliferation: see Table 8) which succinctly sum up the processes involved in CF, and whose expression might well prove to be a reliable marker of CF. However, for a more feasible molecular signature of CF, we decided to reanalyse the raw microarray data of Ogilvie et al. [19], using the RP method as a way of seeing “further down” their data sets for both nasal epithelium, in which they only identified a handful of significantly regulated genes, and bronchial epithelium, which was the tissue which shared more up-regulated genes with our dataset in the preliminary comparison.

Use of the RP statistical method to detect even incremental differential gene expression with a high level of significance allowed us to produce gene lists of comparable sizes for both nasal and bronchial epithelial cell samples from the Ogilvie et al. [19] study. Our analysis identified a large number of regulated genes shared by bronchial and nasal epithelium, partly contradicting the authors’ claim that nasal epithelium is not a good surrogate for the CF airway [19], but also supporting that claim given the greatly reduced fold change of expression shown by these genes in nasal cells (see Additional file 3). Comparing the reanalysed gene lists with our own nasal cell data (see Figure 7) identified 30 genes regulated in all three lists (see Table 9). These genes represent a small putative molecular signature for F508del-CFTR expression in airway epithelial cells. A significant number of these genes are involved in inflammation, defence, and responses to wounding, and a number of them are involved in extracellular signalling and calcium ion binding (see Figure 7C). Construction of an association network (see Figure 7D) shows that all of these genes have been linked by co-expression or co-localization in other studies, or have proven functional relationships with each other and with other genes known to be regulated in CF. The most connected gene in this network is IL1B, an important mediator of the inflammatory response and a known modifier of CF lung disease [57].

### Concluding remarks

In summary, our small-scale microarray study of the CF nasal epithelial transcriptome has generated a list of differentially expressed genes which mostly suggest defects in gene regulation networks related to cell proliferation and cilia biology. Comparison of our data set with previously published studies allowed us to assess the consistency of independent microarray data sets, thus revealing the limitations of such snapshot studies in measuring a system as subtle and dynamic as the transcriptome and suggesting that a molecular signature for CF is likely to be of elevated plasticity. Nevertheless, similarities in pathway and Gene Ontology enrichment between our data set and shared genes from other data sets do give evidence for common gene expression components with elevated functional significance to both primary and secondary cellular responses to F508del-CFTR. The novelty of our approach lies in the new perspectives enabled by the application of new statistical analyses to both new and old data sets, and underlines the importance of public data repositories for high throughput data. This has allowed us to identify a small molecular signature characterizing F508del-CFTR expression in both nasal and bronchial native airway epithelium which we believe is worthy of further investigation. Future studies may be able to refine this signature and test its value as a predictive tool for discriminating between CF and healthy tissue samples. Comparing the signature genes with functional genomics data may also help to clarify cellular responses to CFTR dysfunction in the airway epithelium.

## Abbreviations

CBAVD: Congenital bilateral absence of vas deferens; CF: Cystic fibrosis; CFTR: CF transmembrane conductance regulator; DAVID: Database for annotation, visualization and integrated discovery; FDR: False discovery rate; GO: Gene ontology; GSEA: Gene set enrichment analysis; RP: Rank products (analysis).

## Competing interests

The authors declare that they have no competing interests.

## Authors’ contributions

LAC participated in the design of the study, carried out the experimental work, and drafted the manuscript. LS carried out the microarray data analysis and other statistical analyses. CB participated in sample collection and gathered patient data. MDA participated in the design and coordination of the study and helped to draft the manuscript. All authors read and approved the final manuscript.

## Supplementary Material

Additional file 1 Gene lists from current study (“Up-CF” and “Down-CF”). Click here for file

Additional file 2 **Gene lists from comparable studies presented in Table **3**.** Click here for file

Additional file 3 **Genes regulated between CF and non-CF bronchial and nasal epithelium (reanalysis of**[19]**).** Click here for file

Additional file 4 Gene list from current study with relaxed cutoff (pfp<0.05). Click here for file

Additional file 5 **Collapsed gene lists (from Additional files **3 **and** 4**) used in comparison and generation of Table** 9** and Figure **7**.** Click here for file
